# Low-temperature gas from marine shales: wet gas to dry gas over experimental time

**DOI:** 10.1186/1467-4866-10-10

**Published:** 2009-11-09

**Authors:** Frank D Mango, Daniel M Jarvie

**Affiliations:** 1 Petroleum Habitats, 806 Soboda Ct, Houston, Texas 77079, USA; 2 Worldwide Geochemistry, 218 Higgins Street, Humble, Texas 77338, USA

## Abstract

Marine shales exhibit unusual behavior at low temperatures under anoxic gas flow. They generate catalytic gas 300° below thermal cracking temperatures, discontinuously in aperiodic episodes, and lose these properties on exposure to trace amounts of oxygen. Here we report a surprising reversal in hydrocarbon generation. Heavy hydrocarbons are formed before light hydrocarbons resulting in wet gas at the onset of generation grading to dryer gas over time. The effect is moderate under gas flow and substantial in closed reactions. In sequential closed reactions at 100°C, gas from a Cretaceous Mowry shale progresses from predominately heavy hydrocarbons (66% C_5_, 2% C_1_) to predominantly light hydrocarbons (56% C_1_, 8% C_5_), the opposite of that expected from desorption of preexisting hydrocarbons. Differences in catalyst substrate composition explain these dynamics. Gas flow should carry heavier hydrocarbons to catalytic sites, in contrast to static conditions where catalytic sites are limited to in-place hydrocarbons. In-place hydrocarbons and their products should become lighter with conversion thus generating lighter hydrocarbon over time, consistent with our experimental results.

We recognize the similarities between low-temperature gas generation reported here and the natural progression of wet gas to dry gas over geologic time. There is now substantial evidence for natural catalytic activity in source rocks. Natural gas at thermodynamic equilibrium and the results reported here add to that evidence. Natural catalysis provides a plausible and unique explanation for the origin and evolution of gas in sedimentary basins.

## Introduction

Marine shales release gas under isotherm gas flow at low-temperatures [1]. Gas is released discontinuously, in distinct aperiodic episodes that continue over time. It is nonlinear kinetic behavior resembling chaotic catalysis by transition metals [2]. Trace levels of oxygen suppress gas emission, and gas compositions reflect equilibrium control. The recent disclosure of natural gas at thermodynamic equilibrium and catalytic gas from marine shales similarly constrained, strongly suggests natural catalysis as the source of natural gas [3].

The origin of natural gas remains controversial, however. Many believe that thermal cracking is the source and cite various pyrolysis simulation experiments to support this view [4-14]. Recent hydrous pyrolysis experiments would seem to rule out natural catalytic activity in general [15], and activity by transition metals [16] in particular. This conclusion was based on the premise that any natural catalytic activity that might exist would express itself under hydrous pyrolysis conditions. However, natural activity is a low-temperature phenomenon that is not observed at pyrolysis temperatures (> 300°C) [1].

Here we address catalytic gas generation under open and closed conditions to distinguish it from alternative explanations, desorption and thermal degradation in particular. Gas compositions change very little over time in thermal degradation experiments under open and closed conditions. Methane concentrations remain within a narrow range between 20 and 60% vol (C1-C5) irrespective of kerogen type, temperatures, or reaction conditions [17-21]. Desorption under isothermal gas flow follows first-order kinetics with the lighter hydrocarbons eluting before the heavier hydrocarbons [22], as typically seen in gas chromatography. Both processes should give characteristic compositions over time, easily distinguishable from catalytic generation.

Rates of catalytic reactions are controlled by substrate concentrations at catalytic sites. They can vary in molecular weight if mass transport controls hydrocarbon transit to active centers. This is particularly the case in heterogeneous systems where active sites can be isolated from the hydrocarbon pools surrounding them. Mass transport can then control concentrations at active sites and thus product compositions. Under these circumstances, product compositions can be very different under gas flow where mass transport is active [22] and static conditions where it is not. In our systems, we would expect gas flow to carry higher hydrocarbons to active sites while static conditions will limit these sites to the hydrocarbons in-place. The purpose of this research was to see if there were dramatic differences in the gasses emitted under gas flow and static conditions consistent with catalytic generation as opposed to desorption or thermal degradation.

## Results and Discussion

The kinetics of generation and desorption are different and their products reflect the differences. Desorption is first order and therefore yields characteristic exponential curves over time [22]. Rates are proportional to k_a_* [a], where k_a _is the first order rate constant and [a] is the concentration of hydrocarbon *a *in the rock's kerogen and bitumen. Under isothermal gas flow, the gas-phase ratio of two desorbing hydrocarbons, (a) and (b), will be proportional to k_a_* [a]/k_b_* [b], where (a) and (b) denote concentrations in the effluent gas and [a] and [b] denote concentrations in solution (kerogen and bitumens). If *a *is the lighter hydrocarbon (k_a _> k_b_), the ratio (a)/(b) emerging from the rock will fall exponentially over time as the ratio of their concentrations in solution ([a]/[b]) falls exponentially over time. The ratio (a)/(b) will change with [a]/[b], but the exponential fall over time will not. In first order desorption, the ratio (a)_1_/(b)_1 _at any point in time t_1 _is greater than (a)_2_/(b)_2 _at *dt*: [(a)_1_/(b)_1_]* [(1-k_a_)/(1-k_b_)] = (a)_2_/(b)_2_. This will be true at all points in time, from the onset of desorption where [a] and [b] are high, to infinite time when they are in trace amounts. Thus, the fall in (a)/(b) is *independent *of [a]/[b] and time. It is therefore independent of sample history and preparation. It shall make no difference how much *a *and *b *are lost or retained in sample preparation. Desorption can only be the major source of *a *and *b *if (a)/(b) falls exponentially over time.

The dynamics of generation are different. The concentrations of *a *and *b *in the emerging gas now become proportional to concentrations of precursors that generate *a *and *b*. These could be free hydrocarbons or hydrocarbon appendages to kerogen. The ratio (a)/(b) now becomes a function of the average molecular weight (MW) of the substrates feeding the reaction. If the catalytic rate constants are about equal, (a)/(b) = [S_a_]/[S_b_], where S_a _and S_b _are light and heavier substrates, respectively. Substrate MW will diminish over time as higher MW substrates are converted to lower MW substrates. Thus, [S_a_]/[S_b_] will increase as [S_b_] → [S_a_]. We anticipate two possibilities for catalytic gas generation. With mass transport under gas flow, [S_a_]/[S_b_] should remain relatively constant (steady-state) as higher hydrocarbons are delivered to active sites. Under static conditions where active sites are limited to the hydrocarbons in-place, average MW will decline over time and [S_a_]/[S_b_] will *increase*. Thus, the ratio (a)/(b) should increase under static conditions were mass transport is minimal and remain relatively constant under flow conditions where mass transport is active.

**Figure 1:**
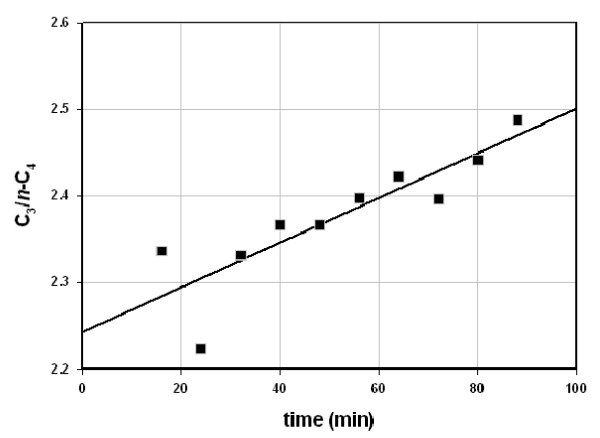
**(Propane)/(n-Butane) ratio emerging from Floyd shale under helium flow at 50°C **[1]. The ratio is ratio of propane and n-butane concentrations (ppm vol) in effluent gas stream at indicated times. The line is the linear regression line, C3/C4 = 2.24 (time) + 0.0026, R^2 ^= 0.76.

We analyzed gas compositions (% mol C_1_-C_5_) from three shales under isothermal gas flow using a procedure described elsewhere [1]. Figure 1 shows the ratio (C_3_)/(*n*-C_4_) *increasing *under He flow at 50°C (Floyd shale). A similar curve obtains from New Albany shale at 100°C, and a slightly declining curve is seen from Mowry shale at 50°C (Table 1). The propane and butane released under isothermal gas flow is therefore *generated *under gas flow. The fact that it occurs at 50°C, is suppressed by oxygen, and is episodic [1], points to catalytic generation, as opposed to thermal generation.

**Table 1:** Gas compositions (% mol) under isothermal He flow over time

	Floyd Shale, 50°C						
time (min)	Methane	Ethane	Propane	i-Butane	n-Butane	i-Pentane	n-Pentane
0	15.92	28.30	23.48	3.57	13.53	6.11	9.10
8	16.14	33.23	24.83	3.24	11.31	4.41	6.84
16	15.53	34.42	25.87	3.42	11.07	3.67	6.02
24	12.16	32.54	27.51	3.95	12.37	4.54	6.93
32	11.34	34.31	27.97	3.61	12.00	4.15	6.63
40	13.02	34.43	27.71	3.40	11.71	3.70	6.03
48	11.36	35.12	28.13	3.48	11.89	3.63	6.40
56	10.35	35.41	28.47	3.54	11.88	4.15	6.21
64	11.58	34.91	28.28	3.31	11.67	4.30	5.95
72	11.42	34.76	28.69	3.60	11.97	3.72	5.84
80	11.08	34.73	28.35	3.50	11.61	4.73	6.00
88	8.76	36.37	29.74	3.47	11.96	3.66	6.04
% var	44	12	12	1	3	12	12
	**New Albany Shale, 100°C**						
**time (min)**	**Methane**	**Ethane**	**Propane**	**i-Butane**	**n-Butane**	**i-Pentane**	**n-Pentane**
0	3.30	29.05	36.68	3.13	17.24	2.67	7.92
4	1.18	37.08	41.00	3.12	13.71	1.39	2.53
8	1.02	34.90	39.13	3.25	15.96	2.11	3.63
12	0.85	34.13	38.09	3.07	16.74	2.43	4.69
18	0.75	33.27	39.63	3.40	16.67	2.19	4.09
21	0.45	27.45	38.69	3.88	18.62	3.50	7.41
34	0.46	26.31	38.24	3.24	18.00	3.58	10.16
45	0.54	26.19	40.16	2.94	18.20	2.92	9.04
55	0.70	25.02	41.61	2.94	18.38	2.73	8.62
65	0.71	23.68	42.29	3.09	18.54	2.86	8.82
% var	71	75	8	2	14	16	109
	**Mowry Shale, 100°C**						
**time (min)**	**Methane**	**Ethane**	**Propane**	**i-Butane**	**n-Butane**	**i-Pentane**	**n-Pentane**
0	9.15	6.84	19.12	7.94	22.71	12.99	21.25
8	4.50	4.34	18.36	11.25	24.02	17.57	19.97
16	3.86	2.95	16.79	12.06	24.43	18.99	20.92
24	5.11	3.16	15.77	11.40	23.29	19.53	21.74
32	-	4.14	16.06	12.88	24.50	20.55	21.87
40	-	-	16.47	13.14	24.34	22.81	23.24
48	-	-	15.97	13.84	24.98	23.06	22.15
56	-	-	15.33	14.44	24.55	23.15	22.52
64	-	-	15.27	14.20	23.92	24.16	22.45
72	-	-	14.92	15.17	23.64	23.71	22.56
80	-	-	11.21	15.06	23.40	26.73	23.60
% var	101	56	26	35	2	68	5

Times are in minutes. Yields: Floyd, 57 μg C_1_-C_5_/g; New Albany, 1.2 mg C_1_-C_5_/g; Mowry, 25 μg C_1_-C_5_/g. % var is variance as % of mean. The dashes indicate no data. Average % variance is 14% in the Floyd data; 42% in the New Albany data; 42% in the Mowry data.

**Figure 2:**
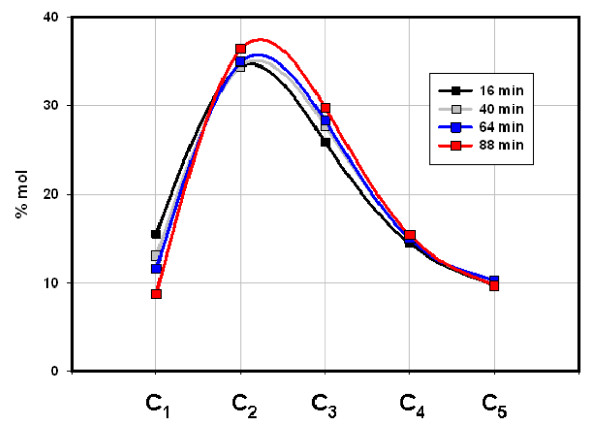
**The distribution of C_1_-C_5 _hydrocarbons emerging from Floyd shale under helium flow at 50°C (Fig. 1)**. C_4 _and C_5 _represent both isomers. 57 μg C_1_-C_5_/g were generated over the course of this experiment.

**Figure 3:**
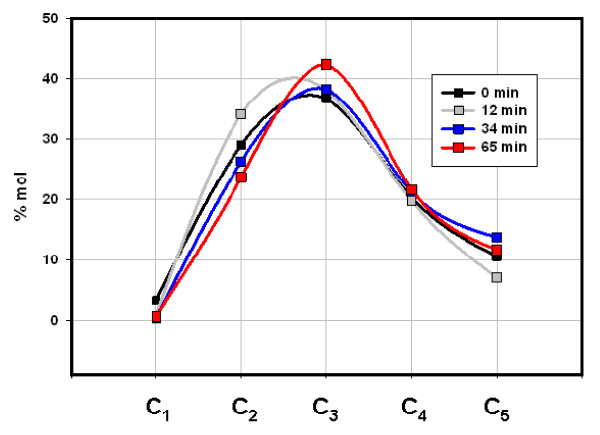
**The distribution of C_1_-C_5 _hydrocarbons emerging from New Albany shale under helium flow at 100°C**. C_4 _and C_5 _represent both isomers. 1.2 mg C_1_-C_5_/g was generated over the course of this experiment. New Albany shale (Dev./Miss.) is side wall core (1025 m) from a well in Union County, KY, Illinois Basin.

**Figure 4:**
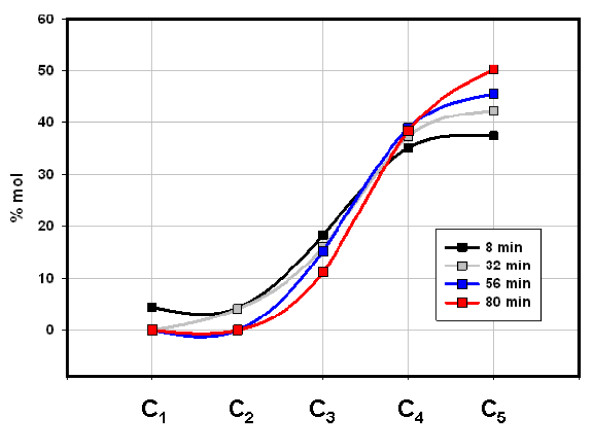
**The distribution of C_1_-C_5 _hydrocarbons emerging from Mowry shale under helium flow at 100°C**. C_4 _and C_5 _represent both isomers. 25 μg C_1_-C_5_/g was generated over the course of this experiment. Mowry shale (Cretaceous) is core (7700 m) from an unknown well in Colorado.

Gas compositions (% vol C_1_-C_5_) changed only moderately as Fig. 1 and Table 1 illustrate. The Mississippian Floyd shale (Black Warrior Basin, MISS) and the Devonian/Mississippian New Albany shale (Illinois Basin, ND) gave similar compositions (Figs. 2 &3) while the Cretaceous Mowry shale (Colorado) gave a distinctly different composition (Fig. 4). Figs. 2, 3, 4 (Table 1) displays three distinct compositions, but each remains relatively constant over time under isothermal gas flow.

**Figure 5:**
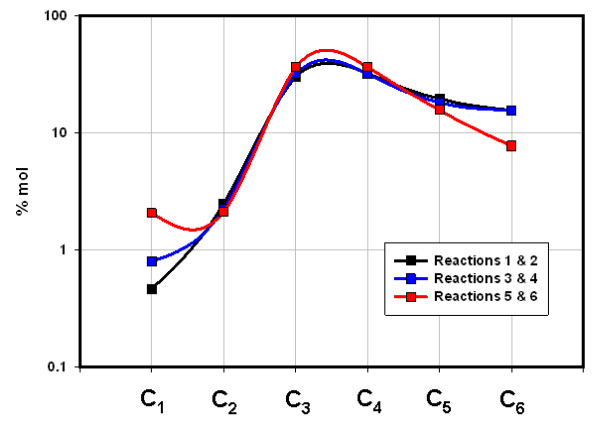
**Concentrations (% mol) of C_1_-C_6 _hydrocarbons generated in six sequential closed reactions, Floyd shale at 50°C (Table 2)**. 'Reactions' refer to the six closed reactions in Table 2. Data points represent average values of two sequential reactions: 'Reactions 1 & 2' is the average value for 1^st ^hr and 2^nd ^hr; 3 & 4 is the average value for 3^rd ^and 4^th ^hr, and so on. C_4 _- C_6 _include all acyclic isomers.

**Figure 6:**
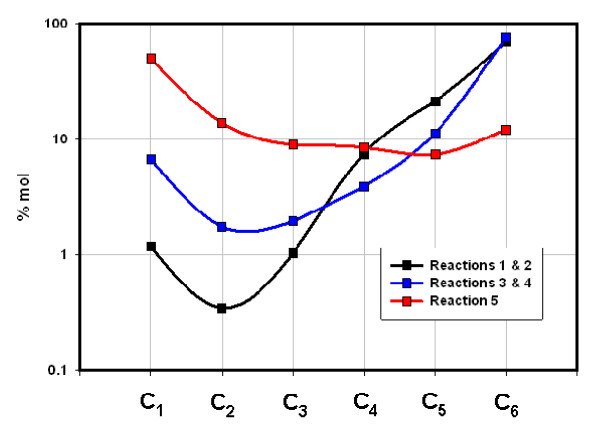
**Concentrations (% mol) of C_1_-C_6 _hydrocarbons generated in five sequential closed reactions, Mowry shale at 100°C (Table 3)**. 'Reactions' refer to the five closed reactions in Table 3. Data points represent average values of two sequential reactions: 'Reactions 1 & 2' is the average value for 1^st ^hr and 2^nd ^hr; 3 & 4 is the average values for 3^rd ^and 4^th ^hr, and Reaction 5 represents the last reaction (24 hr). C_4 _- C_6 _include all acyclic isomers.

To replicate flow conditions without mass transport, samples were subjected to multiple closed reactions. Products were removed between reactions by syringe at ambient temperatures. This generated a series of products representing gas generation unaffected by gas-flow mass transport. The two procedures (gas flow and sequential closed reactions) should give similar products if mass transport makes no contribution to product compositions and substantial differences if mass transport controls product compositions. Seven sequential products from Floyd shale at 50°C are shown in Fig. 5 (Table 2) and six sequential products from Mowry shale at 100°C are in Fig. 6 (Table 3).

**Table 2:** Gas compositions (% mol) in sequential closed reactions, Floyd Shale at 50°C

	1st hr	2nd hr	3rd hr	4th hr	next 19 hr	next 19 hr	% var
**Methane**	0.24	0.88	1.34	0.58	1.21	3.28	91
**Ethane**	3.11	2.77	2.66	2.65	2.82	1.75	8
**Propane**	35.87	35.04	35.46	39.17	41.74	36.75	18
**i-Butane**	11.25	10.39	10.25	9.54	13.05	13.67	24
**n-Butane**	27.05	27.06	27.81	27.46	25.29	26.37	3
**i-Pentane**	11.27	11.18	10.30	9.20	8.33	9.50	14
**n-Pentane**	11.21	12.67	12.19	11.41	7.55	8.67	39
							
**μmol/g, cum**	0.204	0.263	0.304	0.358	0.422	0.445	
							
	**Duplicate Reaction**						
	**1st hr**	**2nd hr**	**3rd hr**	**4th hr**	**next 19 hr**	**next 19 hr**	**% var**
**Methane**	0.31	0.69	-	0.96	1.09	2.59	67
**Ethane**	3.15	2.82	-	2.48	1.95	1.31	23
**Propane**	38.30	36.58	-	34.85	41.58	36.52	17
**i-Butane**	10.40	9.41	-	10.16	11.74	12.68	16
**n-Butane**	26.33	27.19	-	28.18	26.52	27.76	2
**i-Pentane**	10.53	10.53	-	10.49	8.42	9.39	9
**n-Pentane**	10.97	12.78	-	12.87	8.71	9.76	30
							
**μmol/g, cum**	0.268	0.328		0.363	0.441	0.468	

Yields in μmol C_1_-C_5_/g are cumulative. Gas compositions represent gas generated during that period, not cumulative gas composition. The dashed lines represent no data. Total gas yield was 24 μg C_1_-C_5_/g for the first reaction and 26 μg C_1_-C_5_/g for the duplicate reaction. % var is the variance in compositions over the indicated time periods as % of mean. The average % variance for the seven hydrocarbons is 28% for the first reaction and 24% for the duplicate reaction.

The two shales emit entirely different gases under flow and closed conditions with two distinctions outstanding. First, overall compositions remained distinct throughout both procedures. The Floyd product was dominated by ethane under gas flow (Fig. 2) and propane and butane under closed conditions (Fig. 5). Mowry gas under flow conditions (Fig. 4) bears no resemblance to Mowry gas under closed conditions (Fig. 6). Secondly, gas compositions under closed conditions progressed to lighter hydrocarbons over time. The effect was subtle but clear in the Floyd experiment (Fig. 5), and dramatic in the Mowry experiment (Fig. 6). It is definitive evidence against desorption as the source of hydrocarbons in these experiments.

The stark differences between gas flow and closed conditions are also illustrated in data variance. Average variance (% of mean over time) in Floyd data was 14% under gas flow (Table 1) and 28% in closed reactors (Table 2). The average variance in Mowry data went from 33% under gas flow (Table 1) to 740% under closed conditions (Table 3). It is noteworthy that the product in Fig. 4 bears no resemblance to that in Fig. 6 for the same shale at the same temperature under flow and static conditions, respectively.

**Table 3:** Gas compositions (% mol) in sequential closed reactions, Mowry Shale at 100°C

	1st hr	2nd hr	3rd hr	4th hr	next 24 hr	% var
**Methane**	0.77	1.80	11.21	19.89	53.13	2651
**Ethane**	0.27	0.49	3.38	9.33	16.25	783
**Propane**	5.69	3.61	4.50	8.96	12.06	176
**i-Butane**	13.89	11.56	9.98	5.66	2.48	243
**n-Butane**	21.39	15.87	12.48	10.15	7.82	208
**i-Pentane**	30.56	36.72	33.64	26.42	3.90	650
**n-Pentane**	27.41	29.95	24.82	19.59	4.37	488
						
**μmol/g, cum**	0.145	0.171	0.181	0.185	0.203	
						
	**Duplicate Reaction**					
	**1st hr**	**2nd hr**	**3rd hr**	**4th hr**	**next 24 hr**	**% var**
**Methane**	1.80	6.82	23.16	30.24	56.26	1973
**Ethane**	0.49	2.06	5.14	8.95	15.63	569
**Propane**	3.61	2.86	5.40	10.41	10.17	199
**i-Butane**	11.56	6.99	7.03	5.10	2.74	157
**n-Butane**	15.87	11.26	9.08	9.16	6.87	111
**i-Pentane**	36.72	37.10	29.82	20.41	4.24	737
**n-Pentane**	29.95	32.90	20.38	15.74	4.10	649
						
**μmol/g, cum**	0.320	0.370	0.389	0.394	0.410	

Yields in μmol C_1_-C_5_/g are cumulative. Gas compositions represent gas generated during that period, not cumulative gas compositions. Total gas yield was 43 μg C_1_-C_5_/g for the first reaction and 65 μg C_1_-C_5_/g for the duplicate reaction. % var is the variance in compositions as % of mean. The average % variance for the seven hydrocarbons is 743% for the first reaction and 628% for the duplicate reaction.

Mass transport accounts for these differences. Gas flow transports hydrocarbons at rates greatly exceeding diffusion rates [22,23]. We can therefore anticipate a uniform steady-state composition of hydrocarbons at catalytic sites reflecting equivalent rates of hydrocarbon delivery and removal and changing compositions at catalytic sites under static conditions. The relatively uniform distribution of products under gas flow and the changes in hydrocarbon compositions under static conditions are consistent with this interpretation.

The natural progression of wet gas to dry gas is rarely reflected in thermal cracking simulations. This is partly because ethane and propane will not crack at temperatures typically used in thermal cracking experiments [4-15]. Dry gas generation in the laboratory has been reported [27], but only at temperatures above 450°C where these hydrocarbons decompose. With half-lives in the tens of millions [25] to billions of years [26] at temperatures under 200°C, it is very unlikely that thermal cracking could be the source of dry gas in sedimentary basins.

Wet gas to dry gas is replicated here for the first time. It proceeds through *natural *catalytic activity carried from the subsurface. It is *paleoactivity *that has persisted over geologic time as opposed to artificial activity created under laboratory conditions. Shales express activity at ambient temperatures without chemical or thermal activation and lose activity only under artificial conditions, temperatures over 300°C or exposure to oxygen [1]. Marine shales and their counterparts should generate gas in the subsurface as they do in the laboratory, and therefore propose natural catalysis as the source of natural gas in sedimentary basins. It explains high methane concentrations in natural gas, methane enrichment over geologic time, and natural gas at thermodynamic equilibrium [3]. We reject the thermal cracking hypothesis because it cannot explain these observations.

## Conclusion

Hydrocarbons released from Floyd, New Albany, and Mowry shales are products of a natural catalytic process under fluid dynamic control. Desorption of preexisting hydrocarbons makes no significant contribution to the products in our experiments and thermal degradation at these temperatures would be unprecedented and highly unlikely.

The sharp distinctions in yield and compositions between closed reactions and gas flow reactions reflect relatively uniform substrate compositions under gas flow and changing compositions in closed reactions.

These results add to the evidence [1,3] that the natural catalytic activity in carbonaceous sedimentary rocks is the source of natural gas.

## Experimental

The Mississippian Floyd shale and Devonian/Mississippian New Albany shales are described elsewhere [1]. The Cretaceous Mowry shale is whole core (2500 m) from an unknown well in Colorado. Rock-Eval: S1 = 2.61; S2 = 9.33; S3 = 0.15; Tmax = 439. Total Organic Carbon (Leco) = 2.5. The experimental procedures used in sample preparation and product analysis are described elsewhere [1]. The experimental procedure under gas flow is also described in that publication. Closed experiments were carried out in 5 ml glass vials fitted with PTFE/SIL septa purchased from Cole-Parmer (Vernon Hills, IL, USA). Rocks were prepared for analysis by grinding to 60 mesh at ambient temperatures in glove bags under argon, placed in vials under argon, and sealed with open caps fitted with septa. The caps were then wrapped tightly with plastic electrical tape at the vial-screw cap interface to prevent leakage under heating. The vials proved to be leak-proof for the duration of our experiments at 100°C. In sequential experiments, a charged vial containing about 1 gm sample was heated for a time segment, usually 1 hour at constant temperature (± 5°C), then cooled. Product gas was removed by syringe and analyzed as previously described. The vial was then purged of all hydrocarbons by flowing argon in and out of the vial through two needles inserted through the septa (15 min, ~5 ml/min). The purged vials were then reheated for a period of time, usually one hour, and purged again. This procedure was repeated several times. Hydrocarbon compositions in Tables 2 &3 reflect the gas generated in the time segments indicated. The Floyd shale was heated four times at 50°C for one hour, a fifth time for 19 hours at 50°C, and a final time for 19 hours at 50°C. The experiments were duplicated with another sample of Floyd shale (Duplicate Reaction, Table 2). The product from the 3^rd ^heating was inadvertently lost and therefore not analyzed. Duplicate reactions of Mowry shale were carried out in four sequential one-hour reactions at 100°C and a fifth reaction at 100°C for 24 hours (Table 3). Duplicate experiments did not use aliquots of 60 mesh shale. Different samples from the same source were subjected to the same experimental procedures: grinding in argon, sieving, and so forth. The variations in yield and product compositions shown in Tables 2 &3 therefore reflect heterogeneity in samples as well as the variance in our analytical procedure.

## Competing interests

The authors declare that they have no competing interests.

## Authors' contributions

FM formulated theory, and both authors contributed to the experimental work and the final the paper.

## References

[B1] Mango FD Jarvie DM Low temperature gas from marine shales Geochemical Transactions 2009 10 3 19236698 10.1186/1467-4866-10-3 PMC2654466

[B2] Eiswirth M Field J, Gyorgyi L Chaos in surface-catalyzed reactions Chaos in Chemistry & Biochemistry 1993 Ch. 6 World Scientific Publishing Co., River Edge, NJ 141 174

[B3] Mango FD Jarvie DM Herriman E Natural gas at thermodynamic equilibrium: Implications for the origin of natural gas Geochemical Transactions 2009 10 6 19531233 10.1186/1467-4866-10-6 PMC2705366

[B4] Burnham AK Braun RL General kinetic model of oil shale pyrolysis In Situ 1985 9 1 23

[B5] Lewan MD Evaluation of petroleum generation by hydrous pyrolysis Philosophical Transactions Royal Society 1985 315 123 134 10.1098/rsta.1985.0033

[B6] Ungerer P Pelet R Extrapolation of oil and gas formation kinetics from laboratory experiments to sedimentary basins Nature 1987 327 52 54 10.1038/327052a0

[B7] Castelli A Chiaramonte MA Beltrame PL Carniti P Del Bianco A Stroppa F Durand B, Behar F Thermal degradation of kerogen by hydrous pyrolysis. A kinetic study Advances in Organic Geochemistry 1989 Pergamon Press, Oxford 1077 1101

[B8] Horsfield B Disko V Leistner F The micro-scale simulation of maturation: outline of a new technique and its potential applications Geologische Rundschau 1989 78 361 374 10.1007/BF01988370

[B9] Behar F Kressmann S Rudkiewicz JL Vandenbroucke M Experimental simulation in a confined system and kinetic modeling of kerogen and oil cracking Organic Geochemistry 1992 19 173 189 10.1016/0146-6380(92)90035-V

[B10] Lewan MD Engle M, Macko S Laboratory simulation of petroleum formation - hydrous pyrolysis Organic Geochemistry 1993 Plenum Press, New York 419 442

[B11] Muscio GPA Horsfield B Welte DH Occurrence of thermogenic gas in the immature zone - implications from the Bakken in-source reservoir system Organic Geochemistry 1994 22 461 476 10.1016/0146-6380(94)90119-8

[B12] Lewan MD Ruble TE Composition of petroleum generation kinetics by isothermal hydrous and non-isothermal open-system pyrolysis Organic Geochemistry 2002 33 1457 1475 10.1016/S0146-6380(02)00182-1

[B13] Erdman M Horsfield B Enhanced late gas generation potential of petroleum source rocks via recombination reactions: Evidence from the Norwegian North Sea Geochimica et Cosmochimica Acta 2006 70 3943 3956 10.1016/j.gca.2006.04.003

[B14] Lehne E Dieckmann V di Primio R Fuhrmann A Horsfield B Changes in gas composition during simulated maturation of sulfur rich type II-S source rock and related petroleum asphaltenes Organic Geochemistry 2009 40 604 616 10.1016/j.orggeochem.2009.02.004

[B15] Lewan MD Kotarba MJ Wieclaw D Piestrzynski A Evaluating transition-metal catalysis in gas generation from the Permian Kupferschiefer by hydrous pyrolysis Geochimica et Cosmochimica Acta 2008 72 4069 4093 10.1016/j.gca.2008.06.003

[B16] Mango FD Transition metal catalysis in the generation of petroleum and natural gas Geochimica et Cosmochimica Acta 1992 56 553 555 10.1016/0016-7037(92)90153-A

[B17] Voge HH Good GM Thermal cracking of higher paraffins Journal American Chemical Society 1949 71 593 597 10.1021/ja01170a059

[B18] Fabuss BM Smith JO Lait RI Borsanyi AS Satterfield CN Rapid thermal cracking of n-hexadecane at elevated temperatures Industrial and Engineering Chemistry, Product Develoment 1962 1 293 299 10.1021/i260004a011

[B19] Behar F Ungerer P Kressmann S Rudkiewiez JL Thermal evolution of crude oils in sedimentary basins: experimental simulation in a confined system and kinetic modeling Revue de l'Institut Francais du Pétrole 1991 46 151 181

[B20] Horsfield B Schenk HJ Mills N Welte DH An investigation of the in-reservoir conversion of oil to gas: compositional and kinetic findings from closed-system programmed-temperature pyrolysis Organic Geochemistry 1992 19 191 204 10.1016/0146-6380(92)90036-W

[B21] Burkle-Vitzum V Kinetics of alkyl aromatics on the thermal stability of hydrocarbons under geologic conditions Organic Geochemistry 2004 35 3 33 10.1016/j.orggeochem.2003.06.001

[B22] Tokoro Y Misono M Uchijima T Yoneda Y Analysis of thermal desorption curves for heterogeneous surfaces. I. A linear variation of the activation energy of desorption Bulletin of the Chemical Society of Japan 1978 51 85 89 10.1246/bcsj.51.85

[B23] Schaefer RG Weiner B Leythaeuser D Determination of sub-nanogram per gram quantities of light hydrocarbons (C2-C9) in rock samples by hydrogen stripping in the flow system of a capillary gas chromatograph Analytical Chemistry 1978 50 1848 1854 10.1021/ac50035a031

[B24] Mango FD Methane concentrations in natural gas: the genetic implications Organic Geochemistry 2001 32 1283 1287 10.1016/S0146-6380(01)00099-7

[B25] Laidler KJ Sagert NH Wojciechowske BW Kinetics and mechanisms of the thermal decomposition of propane Proceedings Royal Society 1962 A270 242 253 10.1098/rspa.1962.0215

[B26] Laidler KJ Wojciechowske BW Kinetics and mechanisms of the thermal decomposition of ethane. I. The uninhibited reaction Proceedings Royal Society 1961 A260 91 102 10.1098/rspa.1961.0015

[B27] Erdmann M Horsfield B Enhanced late gas generation potential of petroleum source rocks via recombination reactions: Evidence from the Norwegian North Sea Geochimica Cosmochimica Acta 2006 70 3943 3956 10.1016/j.gca.2006.04.003

